# Whole-genome plasma sequencing reveals focal amplifications as a driving force in metastatic prostate cancer

**DOI:** 10.1038/ncomms12008

**Published:** 2016-06-22

**Authors:** Peter Ulz, Jelena Belic, Ricarda Graf, Martina Auer, Ingrid Lafer, Katja Fischereder, Gerald Webersinke, Karl Pummer, Herbert Augustin, Martin Pichler, Gerald Hoefler, Thomas Bauernhofer, Jochen B. Geigl, Ellen Heitzer, Michael R. Speicher

**Affiliations:** 1 Institute of Human Genetics, Medical University of Graz, A-8010 Graz, Austria; 2 Department of Urology, Medical University of Graz, A-8036 Graz, Austria; 3 Department of Internal Medicine I, Hospital Barmherzige Schwestern Linz, A-4020 Linz, Austria; 4 Department of Internal Medicine, Division of Oncology, Medical University of Graz, A-8036 Graz, Austria; 5 Institute of Pathology, Medical University of Graz, A-8036 Graz, Austria

## Abstract

Genomic alterations in metastatic prostate cancer remain incompletely characterized. Here we analyse 493 prostate cancer cases from the TCGA database and perform whole-genome plasma sequencing on 95 plasma samples derived from 43 patients with metastatic prostate cancer. From these samples, we identify established driver aberrations in a cancer-related gene in nearly all cases (97.7%), including driver gene fusions (*TMPRSS2:ERG*), driver focal deletions (*PTEN*, *RYBP* and *SHQ1*) and driver amplifications (*AR* and *MYC*). In serial plasma analyses, we observe changes in focal amplifications in 40% of cases. The mean time interval between new amplifications was 26.4 weeks (range: 5–52 weeks), suggesting that they represent rapid adaptations to selection pressure. An increase in neuron-specific enolase is accompanied by clonal pattern changes in the tumour genome, most consistent with subclonal diversification of the tumour. Our findings suggest a high plasticity of prostate cancer genomes with newly occurring focal amplifications as a driving force in progression.

## Abstract (web summary)

The genomic features of metastatic prostate cancer are beginning to be understood. Here, the authors performed whole genome sequencing of plasma samples from these patients and found a high plasticity of the cancer genomes with newly occurring focal amplifications as a driving force in progression.

## Graphical abstract

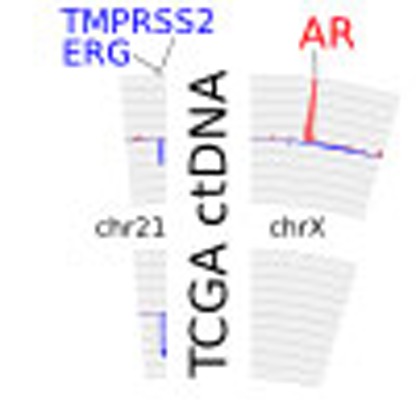

Approximately half of prostate cancer cases harbour recurrent gene fusions involving E26 transformation-specific (ETS) transcription factors^1^. Otherwise, the prostate cancer genomes of early and advanced castration-resistant (CRPC) stages have an overall low somatic point mutation rate compared with other cancers^2^,^3^,^4^,^5^,^6^,^7^,^8^. In contrast, an increase and predictive value of somatic copy-number alterations (SCNAs) in prostate cancer progression was reported^9^ and recent large-scale integrated analyses found that outlying expression coincided with copy-number events^5^,^9^. However, the plasticity and evolution of the prostate cancer genome are mostly unknown.

We applied plasma-Seq^10^ to a panel of 95 plasma samples of clinically annotated patients with metastasized prostate cancer (*n*=43; Supplementary Table 1). Plasma-Seq employs whole-genome sequencing from plasma DNA at a shallow sequencing depth (∼0.1–0.2 ×) to establish tumour-specific SCNAs from circulating tumour DNA (ctDNA) at low costs in real time, that is, in <2 days^10^,^11^,^12^,^13^,^14^. In 14 plasma samples from 10 patients, we performed targeted resequencing of 74 cancer-related genes (Supplementary Table 2) encompassing 620 kb at high coverage (∼850 × (173–1,563)). To infer genomic differences between primary and metastasized prostate cancer, we utilized 493 prostate cancer cases in The Cancer Genome Atlas (TCGA) database, which mainly represent early-stage, primary prostate cancers. Our plasma DNA analyses suggest a high plasticity of prostate cancer genomes with newly occurring focal amplifications as a driving force in progression.

## Results

### Assessment of SCNAs

**Figure 1:**
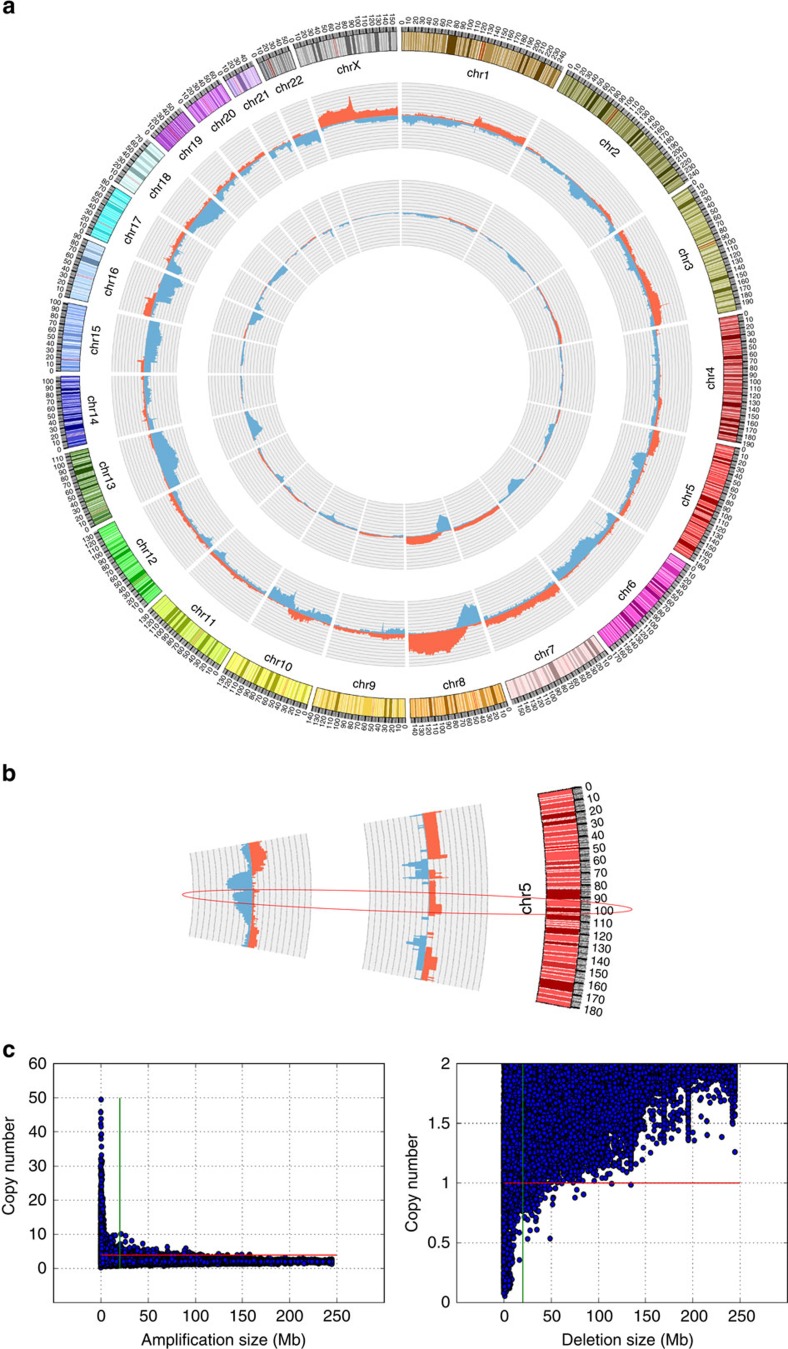
Overall characterization of SCNAs in TCGA and plasma samples. (**a**) Circos plot illustrating the relative frequency of SCNAs (gains in red and losses in blue) of 493 prostate cancer cases from the TCGA database (inner circle) and 95 plasma samples from patients with metastasized prostate cancer (middle circle). The outer circle shows ideograms of the respective chromosomes. (**b**) Partial chromosome 5 circos plot of plasma samples without (inner segment) and with (middle segment) *TMPRSS2:ERG* fusion, the ellipse marks the *CHD1* region. (**c**) Scatter plot of 30 genes commonly involved in amplifications (left panel; green bar: 20 Mb; red bar: copy number of 4) and 42 genes frequently involved in focal deletions (right panel; green bar: 20 Mb; red bar: copy number of 1) derived from 5,737 cases of the TCGA pan-cancer data set demonstrating an inverse relationship between amplification and deletion size, respectively, as well as copy numbers.

Overt SCNAs were observed in 77.5% (*n*=382) and in 77.8% (*n*=74) of TCGA and plasma prostate cancer samples, respectively (Fig. 1a). In a typical TCGA prostate cancer sample, an average of 3.2% (∼98 Mb) of the genome was over-represented and 5.2% (∼162 Mb) lost, whereas in a plasma prostate cancer sample an average of 8.7% (∼268 Mb; Mann–Whitney *U*-test, *P*=1.1 × 10^−17^) and 11.6% (∼359 Mb; Mann–Whitney *U*-test, *P*=7.8 × 10^−7^) of the genome were over-represented and lost, respectively (Fig. 1a). In contrast, when we sequenced plasma DNA from a cohort of male individuals without cancer (*n*=50), the averages of gained and lost regions were merely 0.1% and 0.5%, respectively (Supplementary Fig. 1).

In both the TCGA cases and plasma samples, GISTIC2.0 (genomic identification of significant targets in cancer)^15^ confirmed established prostate cancer SCNAs^9^,^16^, such as the significant gain of 3q and 8q, and the significant loss of chromosomes 8p, 13q, 10q23.31 (harbouring *PTEN*), 17p13.1 (*TP53* locus) and the *TMPRSS2:ERG* fusion associated deletion on chromosome 21q22.3 (ref. 17; Supplementary Fig. 2; Supplementary Data 1). Tumours with ETS fusions (ETS positive) were previously reported to be distinct from those without (ETS negative)^3^,^9^,^18^. Indeed, none of the ETS-positive plasma cases (that is, cases with the *TMPRSS2:ERG* fusion deletion on chromosome 21) showed deletions in *CHD1* at chromosome 5q15-q21.1, which was present in 33% of ETS-negative cases (Fig. 1b). Furthermore, in agreement with earlier observations^3^, we observed loss of the tumour suppressor genes *PTEN* and *TP53* more frequently in ETS-positive cancers (*TP53*: 77% ETS+ versus 42% ETS−; *PTEN*: 66% ETS+ versus 45–54% ETS−).

### Comparison of somatic CNAs in plasma and tissue

As there is rarely a medical reason to biopsy or remove metastases in prostate cancer, referencing our plasma results to any biopsy of metastatic deposits obtained at the time of our blood collection from the same patient represents a challenge and explains why such comparisons were not made in other ctDNA studies dealing with prostate cancer^19^. However, another study reported a remarkable concordance between plasma DNA and matched tissue biopsy, suggesting that plasma DNA analysis provides an accurate status in most patients with prostate cancer^20^. We were able to obtain tissue in eight cases. The tissue was retrieved in relation to our plasma samples within a time interval of 3 months (*n*=1 at the same time (P59); *n*=6 within one month (P2, P29, P33, P40, P112 and P152); *n*=1 within 3 months (P127)) and we therefore considered them to be synchronous plasma and tissue samples. We analysed the tissue with the same approach, that is, whole-genome sequencing with a shallow sequencing depth, and conducted pairwise comparisons of genomic position-mapped profiles. In two cases (P40 and P152), the DNA quality was insufficient for analysis. We observed a strong correlation between tissue and plasma profiles in four of the remaining six pairs (66% (P2, P59, P112 and P127); Supplementary Fig. 3), whereas two cases (P29 and P33) showed a low-correlation coefficient (Supplementary Fig. 3). However, in these two latter samples, there were some SCNAs in the same location in both the plasma and the tumour biopsy, indicating that the plasma samples likely included a branch that shared a common ancestry with the tumour sample. Therefore, ctDNA may contain a gradually shifting conglomerate of DNA released from the tumour lineages present. This emphasizes the fact that a biopsy taken from metastatic prostate disease is just a random, relatively small sample of all tumour events that might not be representative and might not be a major source of ctDNA in the circulation. Similar findings with plasma analyses from patients with prostate cancer were reported earlier by Carreira *et al*.^21^.

### Focal amplifications in prostate cancer genomes

Besides the significant increase of SCNAs between the TCGA and plasma samples, the other most obvious difference was the frequency of focal *AR* gene amplifications (TCGA: 0/493; plasma: 27/43, 62.8%; Fisher exact test, *P*=2.2 × 10^−16^; Fig. 1a), indicating that most of our patients had developed resistance to androgen deprivation therapies (ADTs)^22^,^23^. A focal SCNA is a somatically acquired increase or decrease in copy number of a restricted region harbouring a single or only a few genes^24^. As we had previously observed the emergence of focal amplifications in the plasma of patients with colorectal cancer under anti-EGFR therapy^11^, we hypothesized that focal SCNAs may be an efficient means of adapting to selection pressure in prostate cancer as well.

To test this, we first wanted to define ‘focal' alterations and analysed the size and amplitude of 30 commonly occurring amplified genes, such as *CCND1*, and 42 genes frequently involved in focal deletions, such as *PTEN*^25^ (list of genes in the Methods section), in the 5,737 cases from the TCGA pan-cancer database. We observed an inverse relationship between the size and amplitude of focal copy-number changes (Fig. 1c) and inferred a definition for focal events based on this observation (Methods), which is more stringent than one used in other studies^25^,^26^, to facilitate identification of the gene that is being selected for. Using the TCGA database, we previously showed that SCNAs identified based on this definition correlate with increased gene expression^27^. We confirmed our earlier demonstrations that plasma-Seq reliably detects focal alterations^10^,^11^ by evaluating the prostate cancer cell line VCaP in serial dilutions employing our whole-genome sequencing approach and the CytoScan HD SNP array in parallel. Furthermore, we validated focally amplified genes (*AR*, *FGFR1* and *MYC*) in our plasma samples using quantitative real-time PCR (Supplementary Fig. 4; Methods).

**Figure 2:**
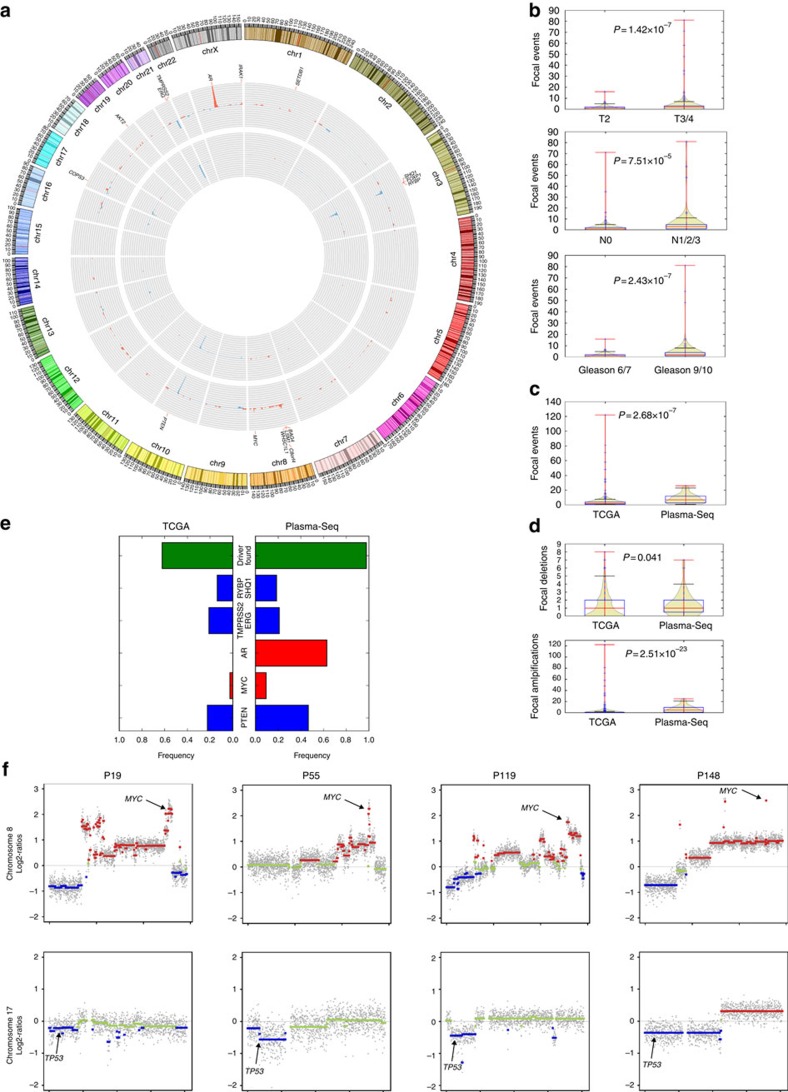
Focal SCNAs in TCGA and plasma prostate cancer samples. (**a**) Circos plot of focal SCNAs in the TCGA database (inner circle) and the plasma samples (middle circle) depicting the relative frequency (*Y* axis limit for amplifications and deletions: 50%) of focal events (gains in red and losses in blue). The outer circle shows ideograms of the respective chromosomes. Target genes of focal SCNAs are annotated if they were observed in >5% of TCGA/plasma samples. (**b**) Boxplot and probability densities of focal SCNA calls for T-stage (top), and N-stage (centre), and Gleason score (bottom). Box comprises data from the first to the third quartile (interquartile range, IQR, in blue) and whiskers (black) extend to the 1.5 × IQR from the box. The median is displayed in red. Probability densities (based on Kernel density estimates) are displayed in ochre and data ranges are displayed in red. *P* values were calculated using Mann–Whitney *U*-tests. (**c**) Boxplot and probability densities of focal copy-number changes in the TCGA and plasma datasets (Whiskers, horizontal lines and statistical test as in **b**). (**d**) Boxplot and probability densities of focal TCGA and plasma samples for focal deletions (top) and for focal amplifications (bottom; Whiskers, horizontal lines and statistical test as in **b**). (**e**) Bar charts depicting the frequency of driver aberrations (green) and relative frequencies of the most frequently observed focal driver amplifications (red) and deletions (blue) in TCGA and plasma samples. (**f**) Co-occurrence of *MYC* amplification and *TP53* losses in patients P19, P55, P119 and P148. Gains (log2-ratio>0.2) are shown in red and losses (log2-ratio less than −0.2) are shown in blue. Green indicates balanced regions.

When we applied our focal SCNA criteria to the 493 TCGA prostate cases, we detected 1,435 focal events in total (∼2.92 focal events per sample) with an average length of 1.94 Mb (range 45 bp to ∼19.7 Mb; s.d. ∼2.69 Mb; Fig. 2a). The mean focal SCNA counts were 1.26 and 3.44 in T2 (*n*=168) and in T3/T4 (*n*=261) patients (Mann–Whitney *U*-test, *P*=1.42 × 10^−7^), respectively, and 2.05 and 5.85 in N0 (*n*=309) and N1 (*n*=65) patients (Mann–Whitney *U*-test, *P*=7.51 × 10^−5^), respectively. Similarly, patients with Gleason scores of 6 or 7 (*n*=259) had a mean focal SCNA count of 1.41 compared with 4.41 in patients with Gleason scores of 9 or 10 (*n*=116; Mann–Whitney *U*-test, *P*=2.43 × 10^−7^; Fig. 2b). Plasma-Seq analyses revealed a total of 594 focal events in 95 samples (∼6.25 focal events per sample; Mann–Whitney *U*-test, *P*=1.45 × 10^−7^ compared with TCGA) with an average length of 2.02 Mb (range 56 kb to 19.4 Mb; SD 2.9 Mb; Fig. 2a,c). When we repeated this analysis using only a single sample per patient to avoid under-counting of events in the same patient, we detected 343 focal events per sample (∼7.98 focal events per sample; Mann–Whitney *U*-test, *P*=2.68 × 10^−7^ compared with TCGA).

We then asked whether the increase of focal events with disease stage originated from amplifications or from deletions. Although the number of deletions per sample increased in our cohort (1.40 focal deletions of one sample per patient) compared with the TCGA (1.14 focal deletions per sample; Mann–Whitney *U*-test, *P*=0.041), disease progression was clearly associated with an increase of focal amplifications (plasma: 6.58 focal amplifications of one sample per patient; TCGA: 1.77 focal amplifications per sample; Mann–Whitney *U*-test, *P*=2.51 × 10^−23^; Fig. 2d). This was an unexpected finding, as the prostate cancer genome was thought to be characterized by relatively few focal chromosomal gains or losses^18^.

We then generated a list of genes known to be frequently amplified in cancer (Methods; Supplementary Data 2) and found that focal amplicons containing such genes were significantly more frequent in plasma than in the TCGA samples (plasma: 2.56 focal amplifications of one sample per patient; TCGA: 0.99 focal amplifications per sample; Mann–Whitney *U*-test, *P*=2.12 × 10^−22^), suggesting that many of these amplicons were involved in tumour evolution. Indeed, we identified an established biological ‘driver' aberration in a cancer-related gene in nearly all cases (42/43 cases, 97.7%; TCGA: 306/493, 62.1%). These included driver gene fusions (*TMPRSS2:ERG*) in 20.9% of patients (TCGA: 20.9%), driver focal deletions such as *PTEN* (46.5%; TCGA: 22.5%) or the 3p13 region (*RYBP* and *SHQ1* locus; 18.6%; TCGA: 13.6%) and driver amplifications such as *AR* (62.8%; TCGA: 0.0%) or *MYC* (9.3%; TCGA: 2.4%; Fig. 2e).

In addition, we found 166 focal amplifications on autosomes in at least two plasma-Seqs or in one plasma-Seq and one TCGA sample that did not contain genes from the known driver list (Supplementary Data 3 and 4). Whether these are passenger or potential driver amplifications remains to be elucidated.

### Amplification of MYC in the prostate cancer genome

The four cases with *MYC* amplification (that is, P19, P55, P119 and P148) all had loss of the 17p13 region (Fig. 2f) and by applying targeted resequencing, we found *TP53* mutations in three cases (that is, one putative splice-site mutation: c.325+5G>A in P19; two stop mutations: p.R196X in P119 and p.R213X in P148), suggesting that these *MYC*-amplified cases did not retain a functional p53 pathway. This is of interest, as we reanalysed the data from the TCGA database and recently found that *MYC*-amplified cases with *TP53* mutations had significantly increased *MYC* expression levels as compared with *MYC*-amplified cases without *TP53* mutations^27^. In contrast, only 12 samples (2.4%) in the TCGA database have *MYC* amplification and none of the eight cases with the available mutation data showed somatic mutations in *TP53*. The four *MYC*-amplified cases were CRPC and hence represent 10.8% (4/37) of cases at this disease stage, which is interesting because of earlier reports that ectopic MYC expression can induce androgen-independent growth^28^. This may have therapeutic consequences, as c-myc-overexpressing cells retaining a functional p53 pathway were shown to respond to etoposide^28^, which would not be applicable in those cases. Furthermore, it is noteworthy that these four cases all had MLL aberrations (P19: *MLL2* frameshift deletion, p.P2552fs; P55: *MLL2* substitution, p.G2344D; P119: *MLL2* substitution, p.M1605L; P148: *MLL3* substitution, p.T1465I). The MLL complex, which is a potential therapeutic target in advanced prostate cancer^29^, interacts with *AR*. Furthermore, *MLL2* mutations were recently shown to occur frequently in lethal CRPC^5^.

### Serial plasma DNA analyses and cancer genome evolution

**Figure 3:**
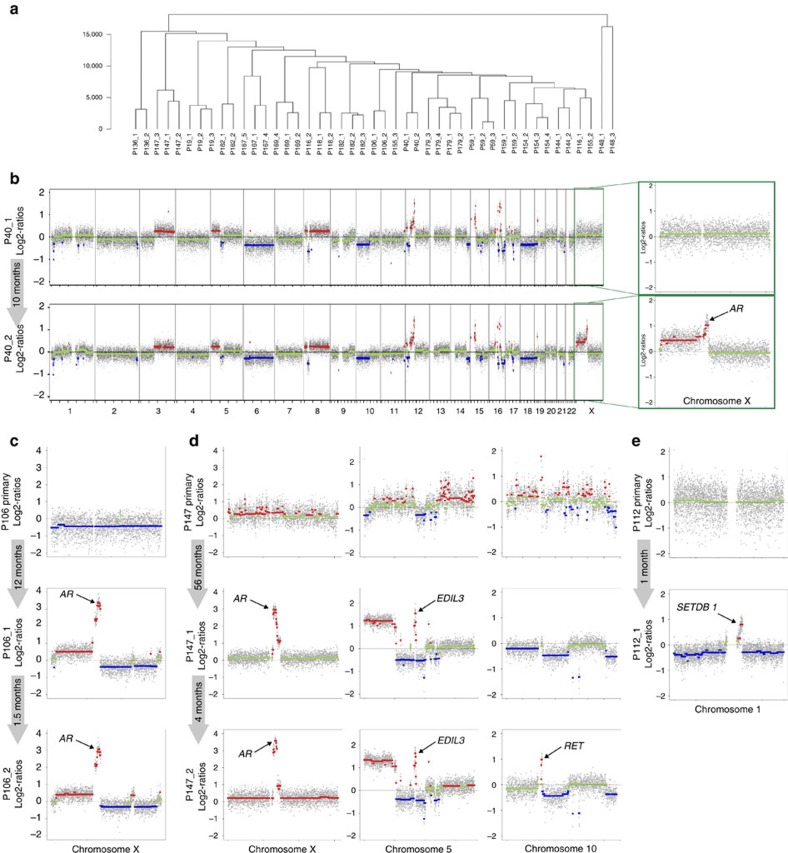
Serial analyses revealed *de novo* occurrence of focal amplifications. (**a**) Hierarchical clustering with serial plasma samples demonstrating that samples derived from the same patient tend to cluster together (Clustering-Manhattan average). (**b**) Genome-wide log2-ratio plots of plasma samples from P40 obtained at a castration-sensitive stage (upper panel) and 10 months later after development of CRPC. The inset illustrates enlarged log2-ratio plots of the X chromosome, the bottom sample shows gain of chromosome X material with the highest copy-number gain on Xq12, the region that harbours the *AR* gene. In this and in the subsequent panels, the grey arrows indicate the time intervals between the sample collections. Copy-number gains are depicted in red and copy-number losses in blue. (**c**) P106's tumour genome developed *AR* amplification within 12 months and an additional amplicon at Xq23-q24 within the subsequent 6 weeks. (**d**) The first plasma sample of P147 had two high-level amplifications, that is, on chromosomes Xq12 (*AR*) and 5q14.3 (*EDIL3*), which had not been observed in the primary tumour. Within the next 4 months, a further amplicon evolved on chromosome 10q11.21 (*RET*). The quality of the analysis of the primary tumour was not optimal due to the fixation conditions of the tissue. The ‘peak' on chromosome 10 in the primary tumour does not involve the *RET* region and is instead most likely an artefact. (**e**) The plasma sample from prostate cancer patient P112 displayed an amplicon on 1q21.3 including *SETDB1*, which had not been present in the analysed part of the primary tumour.

We obtained serial blood samples from 28 patients in addition to primary tumour material from five of these patients (Supplementary Table 1). We performed hierarchical clustering of serial plasma samples with a similar tumour DNA content based on the correlation coefficients, which showed, as expected, that repeated samples from the same patient have higher copy-number similarities than samples derived from other patients (Fig. 3a). For the identification of additional changes as well as relative copy-number shifts, we developed an algorithm for differential copy-number variation-calling from serial copy-number profiles, which we applied to serial plasma samples with high-correlation coefficients, that is, similar ctDNA content (*n*=15 patients). The other patients had various proportions of prostate cancer DNA in the plasma, for example, it was low in one or more of the samples from that patient and usually indicated a good response to the given therapy.

As expected, newly occurring *AR* amplifications were frequently observed (P40, P106 and P147; Methods; Fig. 3b–d) and invariably correlated with clinical signs of progression from CSPC to CRPC. The tumour genome of P106 developed a further amplicon at Xq23-q24 within 6 weeks, harbouring at least 16 genes, none of which have yet been implicated in prostate cancer (Fig. 3c). In patient P147, the first plasma sample collected 56 months after prostatectomy revealed a new high-level amplification on chromosome 5q14.3 in addition to the *AR* amplification (Fig. 3d) with *EDIL3* as the target gene. *EDIL3* encodes a protein involved in angiogenesis and vessel wall remodelling, and its overexpression was reported to accelerate tumour growth by enhancing the vascular formation in hepatocellular carcinoma^30^, implying that *EDIL3* may be a potential target for anti-angiogenic cancer therapy^31^. Within the next 6 months, a further focal amplification emerged on chromosome 10q11.21 (Fig. 3d) with the *RET* oncogene as the target gene. There are several RET targeting agents currently in clinical trials, although they are not yet intended for treating prostate cancer^32^.

In the plasma samples of patient P112, we observed a new amplicon on chromosome 1q21.3 (Fig. 3e), which had not been found in the analysed part of the primary tumour. The amplicon contained three genes (*SETDB1*, *SHC1* and *CKS1B*) with *SETDB1* most likely being the target gene, which is frequently over-represented in prostate cancer^5^. SETDB1 is a histone H3 lysine 9-specific methyltransferase involved in the transcriptional silencing of euchromatic genes and retroelements, and affects cell proliferation, migration and invasion in prostate cancer^33^.

### Evidence for neuroendocrine transdifferentiation

**Figure 4:**
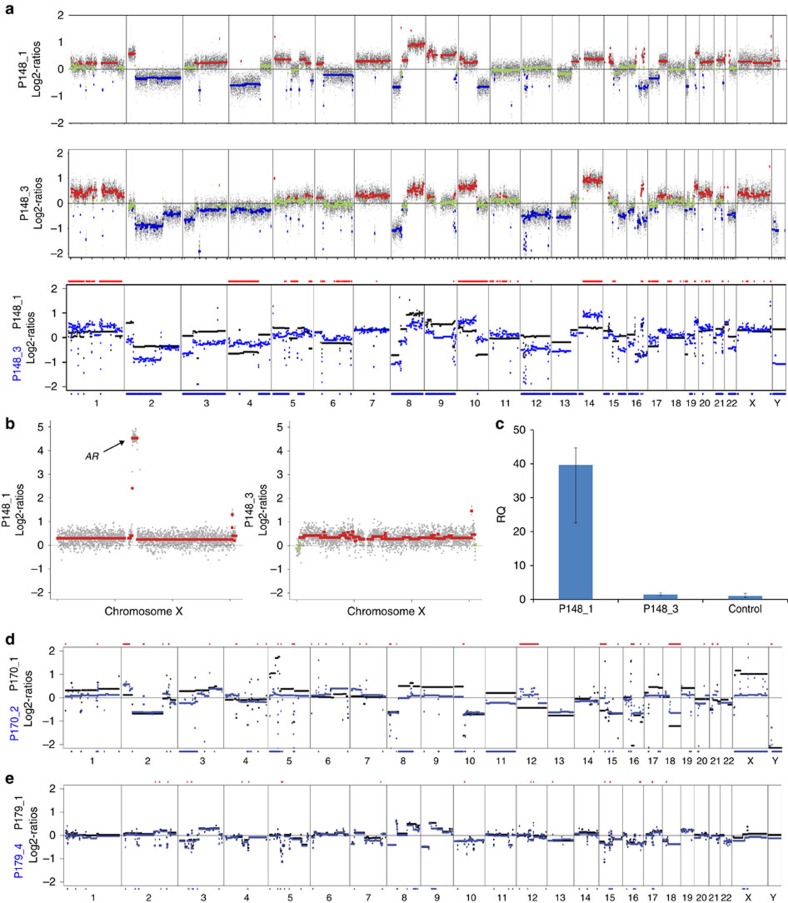
Changing proportions of SCNAs associated with increasing NSE. (**a**) Genome-wide log2-ratio plots of plasma samples P148_1 (upper panel) and P148_3 (centre panel), which was obtained 12 months later. Between these two samples, relative copy-number losses and gains for ∼42.2% and ∼23.3%, respectively, of chromosomal regions (overlay plots in bottom panel; black: first analysis (P148_1); blue: third analysis (P148_3) were observed; regions with different log2-ratios >0.2 are marked with blue and red bars below or above the respective regions). (**b**) Enlarged X-chromosome profiles demonstrating that the *AR* amplification is only present in sample P148_1. (**c**) Confirmation of the *AR* copy-number status by quantitative PCR in duplicates. *Y* axis represents RQ (relative quantity) compared with a pooled male reference DNA sample; error bars indicate minimum and maximum values. (**d**) Plots of P170 illustrating changing chromosomal copy-number patterns over a period of 15 weeks (black: first analysis (P170_1); blue: second analysis (P170_2)). Between the two analyses 26.7% of the genome differs in terms of copy-number status (235 and 590 Mb were gained or lost, respectively, in P170_2 compared with P170_1). Log2-ratios of sample P170_1 have been adjusted to correct for different tumour ratios. Estimated tumour DNA content ratio: 1:4.34 (P170_1/P170_4). (**e**) Comparison between first and last analysed plasma samples from P179 (black: first analysis (P179_1); blue: second analysis (P179_4)). These analyses do not show major changes on the autosomes, but do show loss of the *AR* amplicon (indicated as blue bar below the X chromosome).

A striking observation was changing clonal patterns in cases with increased neuron-specific enolase (NSE). NSE was obtained in some patients, as it may increase and appear as a late manifestation of hormone refractory (AR negative) prostate cancer^34^. At the time of our first analysis, P148 had developed CRPC with a prostate-specific antigen (PSA) of 694.41 ng ml^−1^. Plasma-Seq revealed multiple copy-number changes on the autosomes and gain of the X chromosome with focal *AR* amplification (Fig. 4a–c). Targeted resequencing identified the aforementioned *TP53* mutation (p.R213X) and, in addition, a mutation in *EP300* (p.R1731L) with allele frequencies of 76.9% and 14.4%, respectively. Twelve months after palliative treatment, the disease substantially progressed as indicated by multiple liver and bone metastases. However, the PSA had decreased to 52.0 ng ml^−1^, while the NSE value was >370 ng ml^−1^. At this time, ∼65.5% of the autosomal regions displayed changing proportions of copy-number changes compared with the first sample (Fig. 4a–c). The allele frequency of the *TP53* mutation had increased to 94.8%, whereas the *EP300* mutation was no longer detectable. The X chromosome was still gained, but the *AR* amplicon was no longer detectable as confirmed by quantitative PCR with reverse transcription (Fig. 4c). Using deep sequencing, we did not find any of the common activating *AR* mutations (Methods), which correlated well with the decreasing PSA values.

Similarly, in P170, we observed a decrease of PSA from 16–3.5 ng ml^−1^ and an increase of NSE to 133 ng ml^−1^ over a period of 15 weeks with abiraterone treatment, which was also accompanied by changing the chromosomal copy-number patterns (Fig. 4d) and by massive progression due to liver and lung metastases. The treatment was changed to chemotherapy (carboplatin and etoposide), to which the patient responded very well.

In a further case, that is, P179, the NSE level was already at 59 ng ml^−1^ when we received our first plasma sample. We noted an *AR* amplification (Fig. 4e) at a low PSA value (0.39 ng ml^−1^). During the next 11 weeks, treatment was switched to radiation and chemotherapy so that the selection pressure changed. The PSA level remained low (0.56 ng ml^−1^), but the NSE level increased further to 218 ng ml^−1^. In three further plasma samples, the *AR* amplicon disappeared without additional changes on the autosomes (Fig. 4e). Here the vanishing *AR* amplicon may have been the representative of a perishing clone.

The increasing NSE levels and low PSAs suggested that these cases might have transdifferentiated from an adenocarcinoma to a neuroendocrine prostate cancer (NEPC), which was associated with long-term ADT^34^,^35^. We could not obtain biopsies to confirm this possibility.

## Discussion

We present the data indicating that many changes occur in SCNAs during the progression of metastatic prostate cancer, particularly in focal amplifications, and many potential driver genes were identified in these SCNAs. Altogether, we observed a change in the focal SCNA status in 6 of the 15 cases (40%), for which samples at multiple time points were available and for which many of these new focal changes could affect the clinical management of patients. The mean time interval between these focal alteration changes was 26.4 weeks (range: 5–52 weeks), suggesting that they indicate rapid adaptations to selection pressure. This correlates with a previous report describing clonal dynamics of the *AR* gene in patients with prostate cancer due to adaption to ADT^36^. Our data suggest that it may be practical to use plasma-Seq routinely in advanced prostate cancer to determine such changes in real time.

The SCNA profiles observed are not necessarily the driver of the future metastases at death. But plasma-Seq may lead to a fruitful strategy to find novel pathways during prostate cancer metastases, including rare pathways that are useful even if they are just intermediates in a continuing progression or are ultimately beaten out by an even more lethal sub-lineage.

In contrast to other liquid biopsy methods^21^,^37^,^38^,^39^,^40^,^41^, plasma-Seq is capable of providing valuable information with an unprecedented speed of <48 h at affordable costs^10^. However, our sequencing strategy limits the detection of SCNAs to those occurring at a minimum of ∼5–10% of total ctDNA^10^, but this is a similar resolution as reported elsewhere^21^. Furthermore, the SCNA profiles may be a conglomerate of more than one branch of the evolving tree(s) of tumours, and may hence result in an overestimation of their number that occurs in any particular lineage. If needed, we can complement plasma-Seq by panel sequencing with increased coverage of high-interest genes^10^. However, prostate cancer may be a particularly apt tumour entity for a strategy focusing mainly on SCNAs, since large-scale sequencing efforts for the identification of mutations at the nucleotide level suggested that somatic point mutations in prostate cancer are rare relative to other tumour types^4^,^5^,^6^,^8^. Even the prostate cancer tumour suppressors *TP53* and *PTEN* were described to be commonly altered through copy-number loss rather than point mutations^5^,^9^.

Our data indicate a surprising rapidity of changes in late-stage prostate tumour genomes. Furthermore, our data suggest that an increase in NSE may be an indicator of mesenchymal phenotype transition, that is, transdifferentiated from an adenocarcinoma to a NEPC that could be related to specific detectable changes. Because of new highly potent AR-targeted therapies, the incidence of such treatment-related NEPC was predicted to escalate^35^. At present, it is unclear whether our results question earlier reports that lethal metastatic disease is clonal^42^,^43^,^44^ or whether they are more consistent with subclonal diversification of the tumour based on the stress. An earlier analysis of three chromosomal regions (21q22, 8p21 and 10q23) in ctDNA also described the evidence for independent tumour clones in lethal prostate cancer, albeit not within the context of an NSE increase^21^. However, the primary and metastatic tumours of patients and sequencing with appropriate depth would be needed to truly identify clonal relationships or lack thereof.

In summary, we show that metastasized prostate cancer has a plethora of focal amplifications, many of which span genes known to be a driving force in progression in prostate cancer and other cancers^5^,^9^,^24^,^32^,^45^,^46^,^47^, and some of which may encode previously unrecognized drivers (Supplementary Data 3 and 4). The prevalence of these foci can change rapidly, indicating continual selection. PSA decrease and NSE increase may indicate phenotype transition associated with shifting clonal patterns. The high tumour genome plasticity has significant consequences for clinical trials and our results have tremendous implications for studies testing novel second-generation ADTs.

## Methods

### Summary of clinical characteristics of patients

The study was approved by the Ethics Committee of the Medical University of Graz (approval number 21-228 ex 09/10), conducted according to the Declaration of Helsinki, and written informed consent was obtained from all patients.

At the time of the first blood collection, 6 (14.0%) patients were castration-sensitive (CSPC) and 37 (86.0%) patients were CRPC. The majority of cases (35/43; 81.4%) displayed typical high-grade prostate adenocarcinoma features, five cases (11.7%) were poorly differentiated prostate cancers, one case each (2.3%) had undifferentiated or glandular histology and in one case (2.3%) the histology could not be obtained. No case showed neuroendocrine differentiation or exhibited small-cell neuroendocrine features. In the following are detailed histories of the patients with serial plasma DNA analyses.

*P40*. We did not have access to the primary tumour for patient P40, yet an initial plasma DNA analysis revealed multiple copy-number changes on the majority of autosomes, whereas no copy-number change was observed on the X chromosome (Fig. 3b). Before this therapy, the patient was treated with local radiation. Due to disease progression, treatment was switched to the third-generation LHRH antagonist degarelix^48^. However, despite this therapy switch, progression was noted 10 months later and a repeated plasma analysis revealed that while the changes on the autosomes were the same, there was a focal amplification on chromosome Xq12, which harbours the *AR* gene (Fig. 3b).

*P106*. In P106, there was no *AR* amplification in the primary tumour, yet it was clearly visible in two plasma samples obtained 12 and 13 months later after prostatectomy (Fig. 3c). After diagnosis, treatment was started with the third-generation LHRH antagonist degarelix^48^. Most interestingly, in the <6 weeks between the first and second plasma sample, an additional amplicon evolved at Xq23-q24 (http://genome.ucsc.edu/cgi-bin/hgTracks?db=hg19&position=chrX%3A111225438-117605904), which harbours at least 16 genes. So far, none of these has been implicated in prostate cancer.

*P112*. In the two plasma samples (P112_1 and P112_2) of patient P112, we observed a *de novo* amplicon on chromosome 1q21.3 (Fig. 3e) which had not been present in the primary tumour. The amplicon contained three genes (*SETDB1*, *SHC1* and *CKS1B*) and the most probable target gene is *SETDB1*, which had previously been described as being frequently over-represented in prostate cancer^5^. *SETDB1* is a histone H3 lysine 9-specific methyltransferase involved in the transcriptional silencing of euchromatic genes and retroelements, which is an established oncogene. In prostate cancer, *SETDB1* affects cell proliferation, migration and invasion^33^. During the period between primary diagnosis and obtainment of our plasma sample, the patient was treated with the third-generation LHRH antagonist degarelix.

*P147*. In patient P147, the time period between prostatectomy and the first plasma sample was 56 months. Twenty months after surgery, an increased in PSA levels was noted and treatment with radiation was initiated. Twenty-eight months after diagnosis, the PSA increased again. This was treated for 13 months with the non-steroidal antiandrogen bicalutamide and for the subsequent 4 months, the GnRH-analogue leuprorelin was additionally administered and eventually later the monoclonal antibody denosumab was added due to detection of bone metastases. At the time of further increase in PSA levels, we received the first plasma sample, at which point we noted that novel high-level amplifications developed on Xq12 (*AR*) and on chromosome 5q14.3. A further high-level focal amplification evolved on chromosome 10q11.21, which occurred between collection of the first and second plasma samples (that is, P147_1 and P147_2); the time period between these two samples was 6 months (Fig. 3d). During this time, the patient was treated with chemotherapy, that is, docetaxel.

*P148*. P148 was diagnosed with an adenocarcinoma of the prostate. We obtained our first plasma sample 16 months after the initial diagnosis (Fig. 4a-c) and at this time the patient had clearly progressive disease with increasing metastases to the bone and newly diagnosed lymphadenopathy. Because of the progressive disease (PSA: 694.41 ng ml^−1^), the patient was treated with docetaxel for 7 months. A second plasma DNA analysis during this time (not shown in Fig. 4a–c) confirmed the presence of the high-level *AR* amplification. Five months after the last docetaxel treatment, massive progression with multiple liver and bone metastases was noted, with a PSA level of 52.0 ng ml^−1^ and an NSE value of >370 ng ml^−1^. The patient received palliative treatment with carboplatin and etoposide with an initial partial response lasting 3 months. Thereafter, his disease progressed and he deceased 2 months later.

Patient P179 (Fig. 4d) had an increased NSE level (59 ng ml^−1^) and low a PSA level (0.39 ng ml^−1^) when we received our first plasma sample. At this time, treatment with docetaxel was started. However, the patient did not respond and the level of NSE increased further to 218 ng ml^−1^, while the PSA value remained low (0.56 ng ml^−1^).

*P170*. We received the first blood sample of patient P170 after a disease course of almost 6 years. At this time, the PSA was 16 ng ml^−1^ and treatment was started with abiraterone. Although the PSA decreased to 3.5 ng ml^−1^ over the next 15 weeks, the patient had clinically massive progression due to liver and lung metastases, which was accompanied by an increase of NSE to 133 ng ml^−1^. Another plasma sample showed changing chromosomal copy-number patterns (Fig. 4d) and the treatment was changed to chemotherapy (carboplatin and etoposide), to which the patient responded very well. This was also reflected in subsequent plasma-Seq analyses, demonstrating reduced ctDNA levels and no evidence for persistence of an *AR* amplification.

*P179*. At the time of initial diagnosis, the prostate cancer was already metastasized with infiltration to the rectum. A biopsy revealed a low-grade adenocarcinoma (pT2, G3 and Gleason 5+5) with a PSA value of 15 ng ml^−1^. Treatment was started with leuprolide acetate and bicalutamide. Five months later, metastases to the lymph nodes were detected and another month later bone metastases as well. At this time, we received our first plasma sample. Both PSA (0.39 ng ml^−1^) and testosterone (0.24) were low, but NSE was increased to 59 ng ml^−1^. The treatment was switched to denosumab, palliative radiotherapy and docetaxel. The last blood sample was taken 11 weeks after the first (NSE: 218 ng ml^−1^), at which point the patient showed clear signs of clinically progressive disease.

### Plasma DNA preparation and Plasma-Seq

Blood was drawn (9 ml) into EDTA Vacutainer tubes (BD Biosciences), and 0.225 ml of a 10% neutral-buffered solution containing formaldehyde (4% weight per volume; Sigma-Aldrich, Vienna, Austria) was added immediately after blood withdrawal to prevent cell lysis. Cell-free DNA was isolated from 1 ml of plasma using the QIAamp DNA Blood Mini kit (Qiagen, Hilden, Germany) or the Qiagen Circulating Nucleic Acids kit (CNA; Qiagen). When using the QIAamp DNA Blood Mini kit, 1 ml of plasma was aliquoted to 3 × 333 μl, and DNA extraction was performed on a QIAcube. Extractions using the Qiagen Circulating Nucleic Acids kit (CNA) were performed according to the manufacturer's instructions. For quantification of plasma DNA, we used the Qubit dsDNA HS Assay kit (Life Technologies, Carlsbad, CA, USA). Subsequently, shotgun libraries for plasma-Seq were prepared using the TruSeq Nano DNA LT Sample preparation kit (Illumina, San Diego, CA, USA) with the following exceptions. First, due to the limited amounts of plasma DNA samples, we used 5–10 ng of input DNA. Second, due to the high fragmentation of plasma DNA, we omitted the fragmentation step. Third, for selective amplification of the library fragments that have adaptor molecules on both ends, we used 20–25 PCR cycles. Plasma DNA libraries were quantified and normalized with quantitative PCR, using primers that are complementary to Illumina-specific adaptor sequences (forward: AATGATACGGCGACCACCGAGAT; reverse: CAAGCAGAAGACGGCATACGA). Six libraries were pooled equimolarily and whole-genome sequencing was conducted on an Illumina MiSeq (Illumina) to generate 150 bp single-end reads^10^.

### Tissue DNA preparation and whole-genome sequencing

Formalin-fixed paraffin-embedded (FFPE) tissue samples from primary tumours and biopsies were cut and areas with a high tumour cell infiltration were macrodissected.

Tumour DNA was isolated using the Maxwell 16 FFPE Plus LEV DNA purification kit (Promega, Madison, WI, USA) and quantified using the Qubit dsDNA HS Assay Kit (Life Technologies). The input amount of DNA used for library prep was between 6 and 840 ng. Shotgun libraries for whole-genome sequencing were prepared using the same protocol as for the above-mentioned plasma-Seq method with slight modifications, including a fragmentation step using the Covaris System (Covaris, Woburn, MA, USA) and using 15–25 PCR cycles depending on the input amount of DNA.

### TruSight target enrichment

We performed targeted enrichment of the coding regions of 72 prostate cancer-associated genes as well as the entire genomic regions of the *TMPRSS2* and *ERG* genes, encompassing 620 kb or 309 exons with 14 plasma DNA samples from 10 patients (P2_1, P19_1, P19_2, P19_3, P55_1, P59_1, P106_1, P106_2, P118_2, P119_1, P143_3, P148_1, P148_3 and P151_1). For this purpose, we combined our modified TruSeq Nano DNA LT Sample preparation^10^ with an optimized TruSight Rapid Capture protocol (Illumina). Briefly, 500 ng of an equimolar pool of six shotgun libraries was used for an initial hybridization for 1.5 h at 58 °C. After removal of the unbound fragments, the enriched library was eluted from the capture beads and prepared for a second round of hybridization for 14.5 h to a maximum of 24 h. After the second wash, enriched fragments were eluted and amplified with 12 PCR cycles with Illumina-specific primers from the Rapid Capture kit (Illumina). For PCR cleanup, 45 μl of Sample Purification Beads was used and the final elution volume was decreased to 22 μl. The enriched library pools were checked for quality on a Bioanalyzer (Agilent Technologies, Santa Clara, CA, USA), quantified using quantitative PCR and sequenced on an Illumina MiSeq (Illumina).

Illumina reads were mapped to the human (hg19) genome using bwa mem^49^. Duplicates were marked and reads were realigned using picard and the Genome Analysis Toolkit respectively^50^. SNPs were called using the Unified Genotyper method of the Genome Analysis Toolkit and annotated using annovar^51^.

### TCGA data

TCGA data were downloaded using firehose_get provided by the Broad Institute. SCNA data generated by the Broad Institute on the Affymetrix Genome-Wide Human SNP Array 6.0 from the 2015_02_04 run were used. We used copy-number changes of the following genes for mapping segment sizes and copy numbers of focal amplifications: *MYC*, *CCND1*, *ERBB2*, *CDK4*, *NKX2-1*, *MDM2*, *EGFR*, *MCL1*, *FGFR1*, *KRAS*, *CCNE1*, *CRKL*, *HMGA2*, *TERT*, *PRKCI*, *IGF1R*, *MYCL*, *MYCN*, *CDK6*, *BCL2L1*, *MYB*, *MET*, *JUN*, *BIRC2*, *YAP1*, *PDGFRA*, *KIT*, *PIK3CA*, *MDM4* and *AR*. For focal deletions, we used the following genes: *CDKN2A, CDKN2B, FHIT, WWOX, PTPRD*, *MACROD2*, *PARK2*, *RB1*, *LRP1B*, *PDE4D*, *RBFOX1*, *PTEN*, *CSMD1*, *DMD*, *OPCML*, *NTM*, *ETV6*, *NF1*, *ATM*, *PRKG1*, *PAX5*, *TP53*, *PTPRN2*, *APC*, *NEGR1*, *GPC6*, *RYR2*, *MAGI2*, *CNTNAP2*, *NAALADL2*, *ANKS1B*, *PARD3B*, *SNTG1*, *CDH13*, *DLG2*, *SDK1*, *MAP2K4*, *DSCAM*, *TMPRSS2*, *ERG*, *SMAD4* and *DCC* (for details see text). Analyses of the TCGA and plasma-Seq data can be accessed via IPython notebook at https://github.com/PeterUlz/FocalAmplifications/blob/master/FocalAmplifications_Prostate_methods.ipynb and Supplementary Software 1.

### GISTIC2.0

We applied GISTIC2.0 (version 2_0_21)^15^ to the TCGA data as well as to the plasma-Seq data using the hg19.mat reference file, a confidence interval of 0.95 and with the gene-gistic and the broad event detection functions off.

### SCNA calling from low-coverage whole-genome sequencing

Copy-number segments of plasma samples were identified using a depth-of-coverage algorithm^10^. In brief, we aligned reads against the human hg19 genome (where the Pseudo-autosomal region of the chromosome Y has been masked) using bwa^49^ and counted reads in 50,000 bins of an average length of ∼56 kbp, where each genomic bin contains the same amount of mappable positions (as determined by *in silico* analysis). Bin boundaries for synthetic 150 bp reads using Burrows-Wheeler Aligner (BWA) for alignment are available on request from the authors. Raw read counts were normalized by the total number of read counts obtained and further smoothed by Locally Weighted Scatter-plot Smoother (LOESS) according to the GC content^52^. Moreover, we normalized resulting bin counts by average read counts of cell-free DNA of 10 non-tumour controls. Resulting read counts were segmented using both circular binary segmentation, and gain and loss analysis provided by the CGHWeb framework^53^. This algorithm calls gains and losses, however, amplifications are easier to detect than deletions since there is no upper bound on the copy number and thus they might even be found in low tumour fractions.

Similarly, we identified SCNAs in tumour samples; however, GC-normalized bin counts were normalized by mean bin counts of low-coverage whole-genome sequencing from constitutional DNA rather than cell-free DNA.

### SCNA calling from the TCGA data set

SCNA data from the TCGA were derived from Affymetrix SNP arrays (Affymetrix Genome-wide Human 6.0) and precomputed segmented log2-ratios were used for the analyses. The tumour fraction in TCGA samples is usually higher and less variable than in plasma samples. Therefore, TCGA SCNA data should be more sensitive and should detect more SCNAs than plasma-Seq, which depends on the ctDNA allele fraction. This suggests that plasma analyses underestimate SCNAs as compared with the TCGA.

### Summary of genomic events

SCNAs were identified by analysing log2-ratios of copy-number segments. We called gains at a log2-ratio threshold of 0.2 and losses at a log2-threshold of −0.2. Copy-number aberrant samples were classified as such if either the cumulative length of amplifications or the cumulative length of deletions was 3 s.d.'s higher than that of the controls. To summarize copy-number variation events, we applied GREVE^54^.

Likewise, a summary of focal amplifications from plasma-Seq and the TCGA was generated using GREVE and plotted using the circos^55^ package.

### Hierarchical clustering

Hierarchical clustering was performed in R by employing the fastcluster package. A distance matrix using Manhattan distances was calculated and clusters were merged using the average method in the hclust function.

### Differential SCNA calling

To detect additional SCNAs throughout the genome of subsequent samples, we compared plasma-Seq data of these samples. We focused on subsequent plasma samples with high-correlation coefficients (Spearman correlation coefficient>0.85) since high variability in tumour content may lead to biases.

Because the relative amount of tumour DNA may vary between different time points, we first searched for a tumour ratio between subsequent samples by calculating the sum of squares error of the segmented log2-ratios of each sample for tumour ratios from 0 to 10 at steps of 0.01. As an approximation for tumour content variation, we used the ratio having the least sum of squares error and recalculated the log2-ratios of the subsequent sample by subtracting the log2-ratios of the first sample and multiplying by the most appropriate tumour content ratio.

### Definition of cancer driver genes in focal amplifications

To establish the role of known cancer genes, we searched amplicons that had to contain at least one gene from the following categories: (a) 90 tyrosine kinases expressed in humans^47^; (b) 77 genes obtained from a census of amplified and overexpressed human cancer genes with the evidence of involvement in human cancer development^24^; (c) cancer type-specific genes, which had recently been shown to be deregulated by sequence amplification and likely to be involved in the pathophysiology of prostate^5^,^9^,^45^,^46^ cancer; and (d) potentially druggable targets^32^ (summarized in Supplementary Data 2).

### Focal amplification calling in plasma samples

From segmented copy-number data, we identified focal events using the following criteria for amplifications:

1. Segment should be <20 Mb;
2. Log2-ratio must be >0.2;
3. Segment should contain a gene, but not >100 genes;
4. Log2-ratio must be 0.2 higher than weighted mean of the log2-ratios of neighbouring 20 Mb on both the sides if it contains a known tumour driver gene;
5. Log2-ratio must be 0.58 higher (Log2-ratio of 0.58 translates to about three copies) than weighted mean of the log2-ratios of neighbouring 20 Mb on both the sides if it does not contain a known tumour driver gene;
6. Segment should not contain segmental duplications in >50% of its size;
7. Segment should not overlap with known entries in DGVar.

For focal deletions, we used the following criteria:

1. Segment should be <20 Mb;
2. Log2-ratio must be lower than −0.2;
3. Segment should contain a gene known to be affected by deletions;
4. Segment should contain a gene but not >100 genes;
5. Log2-ratio must be 0.2 lower than weighted mean of the log2-ratios of neighbouring 20 Mb on both the sides;
6. Segment should not contain segmental duplications in >50% of its size;
7. Segment should not overlap with known entries in DGVar.

Focal identification was performed using R and can be accessed along with the sample data via in IPython notebook format (see Data availability).

### Control experiments for the detection of focal SCNAs

We then tested the resolution limits for the detection of amplifications by plasma-Seq. Applying the definition as outlined in the text, 66 focal events were identified in the undiluted VCaP DNA with highly variable amplitudes (range: 3.7 to −4.7) including: *E2F3*, *MYC*, *STK11* and Xq12 harbouring the *AR* gene, which is in agreement with previous characterizations of this cell line^56^. We then evaluated these focal events in serial dilutions in parallel by plasma-Seq and Affymetrix CytoScan HD SNP array analyses and found high-correlation coefficients for the log2-ratios (Spearman correlation), which as expected, decreased with increasing dilution, since log2-ratios of balanced copy numbers mostly represent noise (Supplementary Fig. 3). Furthermore, we validated copy numbers of three focally amplified genes (*AR*, *FGFR1* and *MYC*) using quantitative real-time PCR.

### TaqMan copy-number assay

To validate the copy-number status of *AR*, *MYC* and *FGFR1*, we used commercially available TaqMan Copy Number Assays from Life Technologies (IDs *AR*: Hs04511283_cn, *MYC:* Hs02602824_cn and *FGFR1:* Hs06222764_cn) containing two primers and a FAM dye-labelled MGB probe. The TaqMan Copy Number RNAseP Reference Assay including a VIC dye-labelled TAMRA probe was used as an endogenous reference gene for the calculation of relative of copy numbers. PCR was set up in duplications according to the manufacturer's instructions. In brief, 1 ng of input DNA was mixed with 5 μl of the TaqMan Genotyping Master Mix and 0.5 μl of both, the gene-specific copy-number assay and the reference assay. After an initial activation at 95 °C for 10 min, the reactions were amplified in 40 cycles (denaturation sot 95 °C for 15 s and annealing /extension at 60 °C for 1 min). Copy-number quantification was performed using the Applied Biosystems CopyCaller Software by relative quantification (RQ) using the comparative CT (ΔΔCT) method.

### Deep sequencing of common activating AR mutations

Using deep sequencing, we investigated the following common activating *AR* mutations (that is, V715M, W741L, W741C, H874Y, T877A, T877S, M895T and M895V; Androgen Receptor Gene Mutations Database (http://androgendb.mcgill.ca)^57^) in exons 4, 5 and 8 in plasma samples from patient P148. Target-specific primer sequences were as follows: AR_V715_F: ATGTCCTGGAAGCCATTGA and AR_V715_R: ATGCTCCCACTTCCCTTTTC; AR_W741_F: CAACCCGTCAGTACCCAGAC and AR_W741_R: TCCTGGAGTTGACATTGGTG; AR_876-896_F: AGGCCACCTCCTTGTCAAC and AR_876-896_R: TTTCCCAGAAAGGATCTTGG; AR_T878_F: CACCTCCTTGTCAACCCTGT and AR_T878: TTTCCCAGAAAGGATCTTGG.

### Correlation analysis of plasma and tissue samples

Pearson correlation coefficients were computed to correlate plasma and corresponding tissue samples by using the cor function in R on segmented log2-ratios of autosomal chromosomes. Sex chromosomes were left out since the controls for tissue samples came from both males and females, and thus introduced bias into their copy number.

### Detection of focal amplifications by whole-genome sequencing

VCaP has several large and focal copy-number changes that were equally identified by both approaches (Supplementary Fig. 3a). These are reflected in the high-correlation coefficients for the log2-ratios (Spearman correlation) which, as expected, decreased with increasing dilution, since log2-ratios of balanced copy numbers most often represent noise (Supplementary Fig. 3b). Although we observed a close correlation of copy numbers between the two approaches, plasma-Seq had an increased dynamic range (Supplementary Fig. 3c), suggesting that copy-number changes may be easier to identify with whole-genome sequencing than with SNP arrays. The focal AR amplification was still detectable in the 5% dilution (Supplementary Fig. 3d). We also validated copy numbers of three focally amplified genes (*AR*, *FGFR1* and *MYC*) in our plasma samples using quantitative real-time PCR, further demonstrating the reliability of plasma-Seq (Supplementary Fig. 3e).

### Data availability

All sequencing raw data have been deposited at the European Genome-phenome Archive (EGA; http://www.ebi.ac.uk/ega/), which is hosted by the EBI, under the accession number EGAS00001001018. Focal identification analysis in R can be accessed along with the sample data via the following URL in IPython notebook format: https://github.com/PeterUlz/FocalAmplifications/tree/master/Focal_amplifications_in_R.ipynb. All remaining data is contained within the paper and Supplementary Information files or available from the author upon request.

## Additional information

**How to cite this article:** Ulz, P. *et al*. Whole-genome plasma sequencing reveals focal amplifications as a driving force in metastatic prostate cancer. *Nat. Commun.* 7:12008 doi: 10.1038/ncomms12008 (2016).

## Supplementary Material

Supplementary Information Supplementary Figures 1-4 and Supplementary Tables 1-2.

Supplementary Data 1 Summary of GISTIC analyses.

Supplementary Data 2 List of cancer driver genes associated with amplifications based on the following references: 1-7

Supplementary Data 3 List of focally amplified regions called in at least two samples of ctDNA (one sample per patient) and TCGA samples, which do not contain a gene listed in Supplementary Data 2.

Supplementary Data 4 List of genes within the identified regions in Supplementary Data 3.

Supplementary Software 1 Focal identification analysis in R and sample data
