# Supplementary material for: Whole-genome plasma sequencing reveals focal amplifications as a driving force in metastatic prostate cancer
Source: Nat Commun. 2016 Jun 22;7:12008. doi: 10.1038/ncomms12008 (PMC4917969; doi:10.1038/ncomms12008)
Supplement: Supplementary Information — Supplementary Figures 1-4 and Supplementary Tables 1-2. [file ncomms12008-s1.pdf]

Supplementary Figures

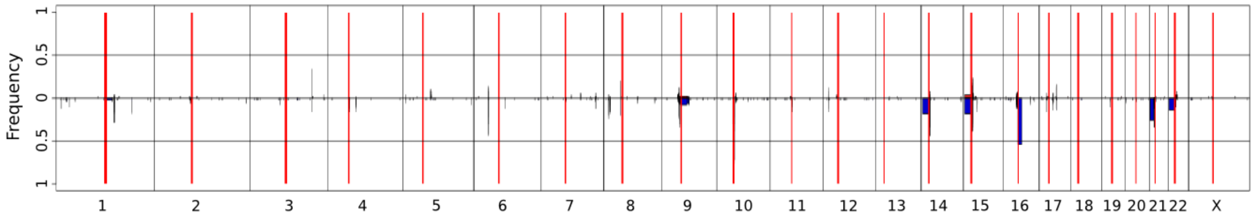

Supplementary Figure 1

Plasma-Seq conducted with blood from male individuals without cancer.

Copy number patterns established from plasma samples of male individuals without cancer (*n*=50). Chromosome positions are indicated along the X-axis, the Y-axis represents relative abundance of respective aberrations in percentage. The averages of gained and lost regions were 0.1% and 0.5% after removing centromeric regions (indicated as red bars), respectively, which were either homologue regions in the vicinity of repetitive regions (e.g. centromeres) or likely constitutional CNVs present in the germline.

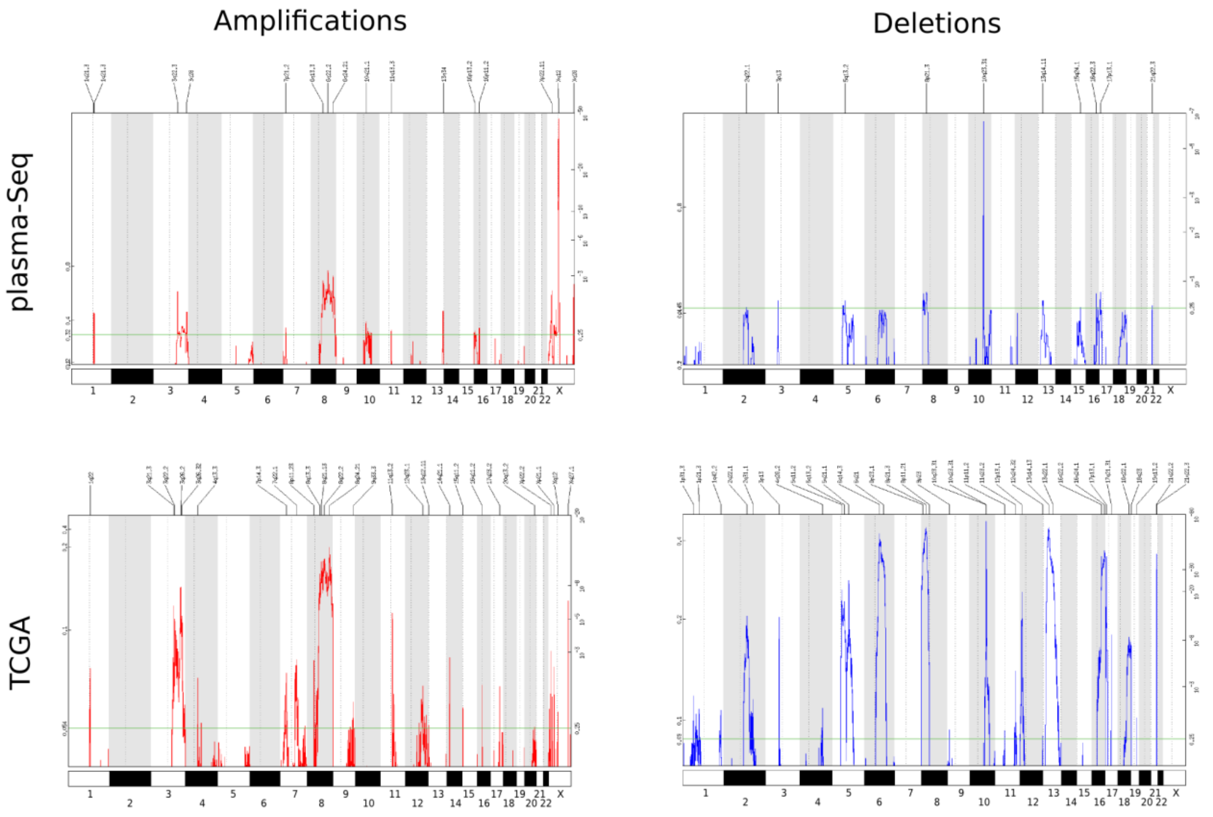

Supplementary Figure 2

GISTIC (genomic identification of significant targets in cancer) analyses.

GISTIC analyses of the TCGA and plasma samples: Amplifications are illustrated in red and deletions in blue. Chromosome positions are indicated along the X-axis. Significance thresholds are indicated by the green lines, the statistical significance of the aberrations is displayed as false discovery rate (FDR) q values on the Y-axis.

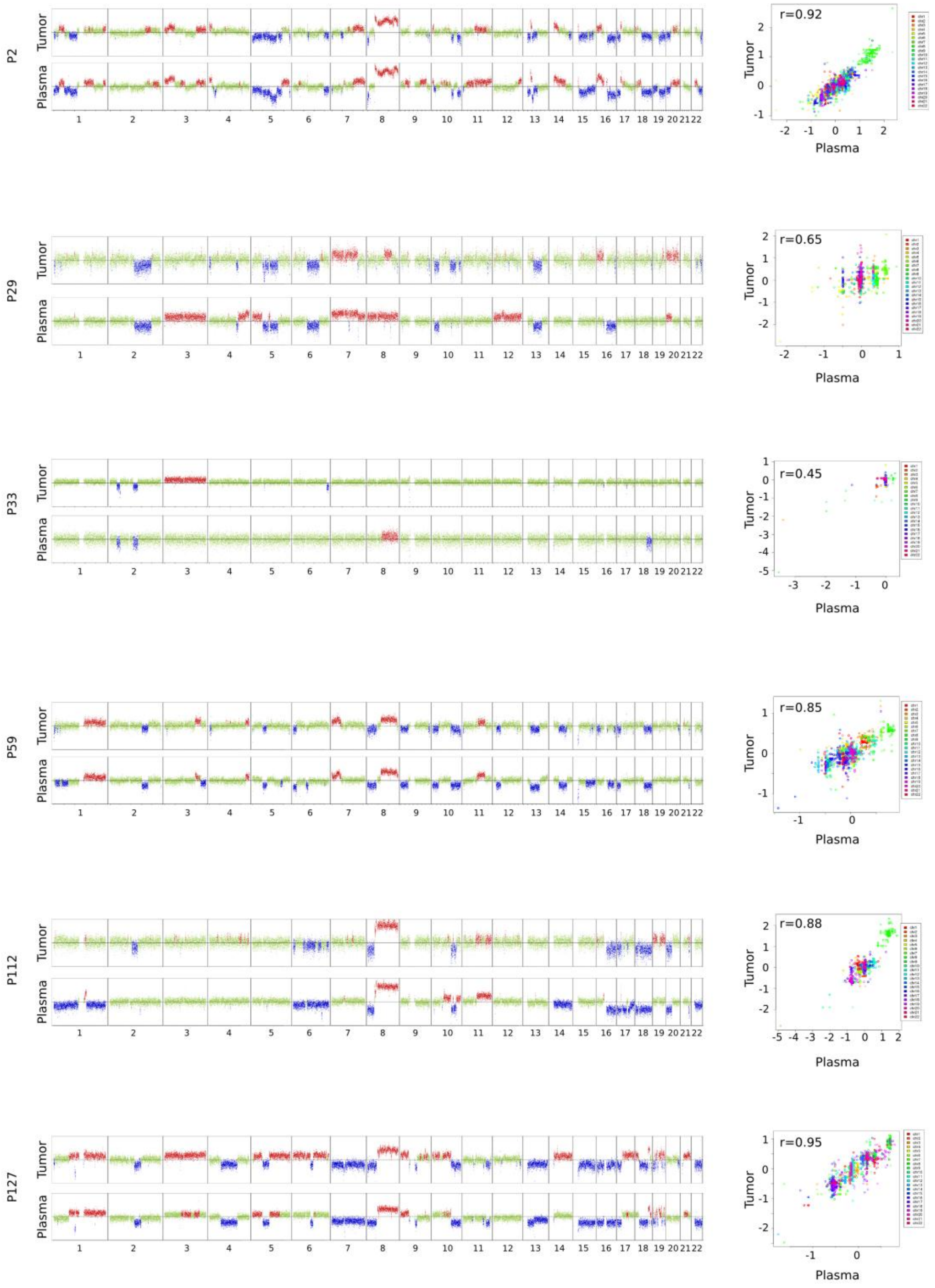

Supplementary Figure 3

Head to head comparisons between plasma and tissue samples.

A pairwise comparison of genomic position-mapped profiles between plasma and tissue samples revealed strong correlations in cases P2 (Pearson correlation coefficient: 0.92), P59 (Pearson correlation coefficient: 0.85), P112 (Pearson correlation coefficient: 0.88), and P127

(Pearson correlation coefficient: 0.95), and low correlations in cases P29 (Pearson correlation coefficient: 0.65) and P33 (Pearson correlation coefficient: 0.45).

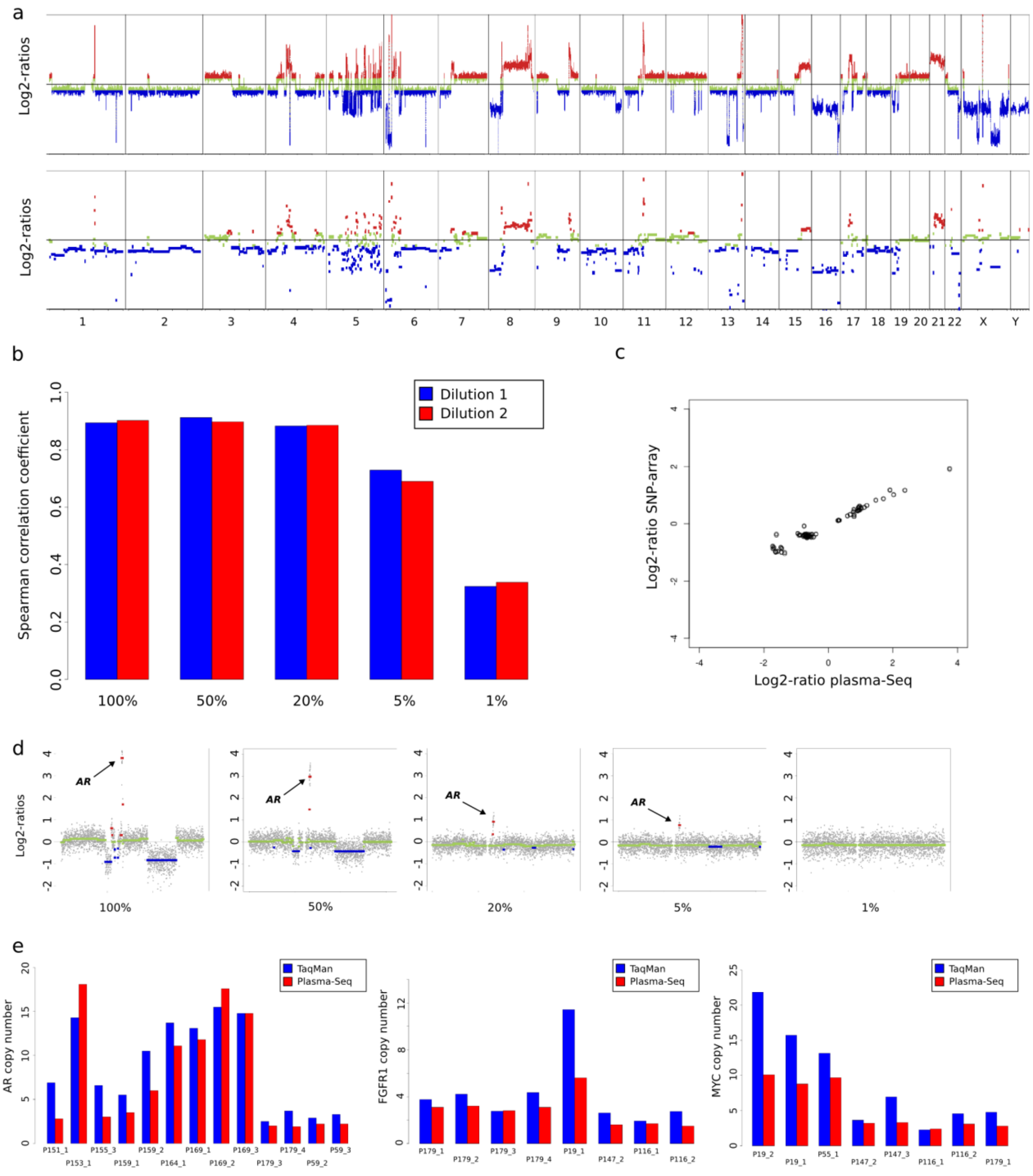

Supplementary Figure 4

Definition of focal SCNAs and detection limits.

(a) Copy number profiles obtained with undiluted VCaP DNA employing the CytoScan HD SNP-array (top) and whole-genome sequencing of VCaP (bottom).

(b) Correlation coefficients (Spearman correlation) obtained by comparison of log2-ratios of focal events identified in undiluted VCaP DNA between whole-genome sequencing and the CytoScan HD SNP-array of two biological replicates (shown in blue and red, respectively) with various dilutions of cell line VCaP.

(c) Scatter plot with copy numbers (inferred from log2-ratios) obtained with CytoScan HD SNP-array (Y-axis) and whole-genome sequencing (X-axis), of focal events identified from whole-genome sequencing of undiluted VCaP DNA, illustrating the correlation and also the higher dynamic range of whole-genome sequencing.

(d) Detection of the AR amplicon in cell line VCaP undiluted (100%) and in a dilution series (50%, 20%, 5%, and 1%).

(e) Validation of copy numbers of three focally amplified genes (*AR*, left panel; *FGFR1* center panel; *MYC* right panel) using quantitative real-time PCR.

Supplementary Tables

Supplementary Table 1

Summary of clinical data of our prostate cancer patient cohort. WNR= within normal range; NSE < 17: normale range; NA: not available

Sample ID	Age (years)	Histology	CRPC/ CSPC	PSA (ng/ml)	NSE (ng/ml)	Therapy
P2_1	73	Adenocarcinoma	CSPC	19,1	WNR	/
P10_1	67	Adenocarcinoma	CRPC	10,4	WNR	ADT
P19_1	69	Adenocarcinoma	CRPC	444,1	WNR	ADT
P19_2				397,3		
P19_3				131,5		ADT
P29_1	73	poorly differentiated PC	CSPC	394,0	WNR	/
P33_1	75	poorly differentiated PC	CSPC	58,3	WNR	ADT
P40_1	83	poorly differentiated PC	CSPC	773,7	WNR	ADT
P40_2			CRPC	656,0		
P55_1	63	Adenocarcinoma	CRPC	49,2	WNR	ADT, Chemotherapy, Abiraterone
P59_1	62	Adenocarcinoma	CRPC	6471,1	WNR	NA
P59_2				7532,5		
P59_3				6863,3		
P65_1	73	Adenocarcinoma	CRPC	303,0	WNR	ADT, Chemotherapy
P65_2				NA		
P98_1	/	/	CRPC	35,6	WNR	RTX
P106_1	79	Adenocarcinoma	CRPC	761,5	WNR	ADT
P106_2				NA		
P112_1	80	Adenocarcinoma	CSPC	146,9	WNR	No therapy applied
P112_2				31,5		ADT
P116_1	85	Adenocarcinoma	CRPC	506,7	WNR	ADT
P116_2				2489,0		Abiraterone
P118_1	68	Adenocarcinoma	CRPC	1604,0	WNR	ADT
P118_2				1689,0		
P119_1	67	poorly differentiated PC	CRPC	161,4	WNR	ADT
P120_1	68	Adenocarcinoma	CRPC	867,7	WNR	ADT, Chemotherapy
P125_1	61	Adenocarcinoma	CRPC	58,8	WNR	ADT
P127_1	57	Adenocarcinoma	CRPC	2,8	WNR	ADT
P136_1	75	Adenocarcinoma	CRPC	208,2	WNR	ADT
P136_2				254,9		

P137_1	77	Adenocarcinoma	CSPC	5,5	WNR	ADT
P140_1	63	Adenocarcinoma	CRPC	593,0	WNR	ADT, Chemotherapy, Abiraterone
P143_1	45	Adenocarcinoma	CRPC	0,0	NA	ADT, Chemotherapy
P143_2				0,2		Abiraterone
P143_3				8,7	>370	Chemotherapy
P144_1	68	Adenocarcinoma	CRPC	34,2	WNR	ADT, Chemotherapy
P144_2				33,0		Abiraterone
P147_1	60	Adenocarcinoma	CRPC	205,0	NA	ADT, Chemotherapy, Abiraterone
P147_2				263,0		
P147_3				863,0	>370	

P148_1	63	Adenocarcinoma	CRPC	694,0	NA	ADT, Chemotherapy
P148_2				48,0		Chemotherapy
P148_3				52,0	>370	
P151_1	66	Adenocarcinoma	CRPC	471,0	WNR	ADT, Abiraterone
P151_2				0,7		
P151_3				0,0		
P152_1	69	Adenocarcinoma	CRPC	NA	WNR	ADT, Chemotherapy
P153_1	68	Adenocarcinoma	CRPC	531,3	WNR	ADT
P154_1	51	Adenocarcinoma	CRPC	NA	WNR	ADT
P154_2				67,7		
P154_3				201,4		Abiraterone, Enazalutamide
P154_4				337,7		Chemotherapy
P155_1	67	undiff.	CRPC	134,0	WNR	ADT, Chemotherapy, Abiraterone, Enzalutamide
P155_2				124,6		
P155_3				NA		
P156_1	59	poorly differentiated PC	CRPC	6,1	WNR	ADT, Abiraterone, Chemotherapy
P156_2				3,2		
P156_3				NA		
P156_4				2,2		
P156_5				NA		
P158_1	61	Adenocarcinoma	CRPC	43,1	WNR	Abiraterone
P158_2				9,2		
P158_3				19,6		Enzalutamide
P158_4						
P159_1	54	Adenocarcinoma	CRPC	319,5	WNR	ADT, Abiraterone
P159_2				599,0		Chemotherapy, Enzalutamide
P162_1	72	Adenocarcinoma	CRPC	NA	WNR	ADT, Chemotherapy
P162_2				69,9		Abiraterone
P164_1	69	Adenocarcinoma	CRPC	292,7	WNR	RTX, Chemotherapy
P165_1	49	Adenocarcinoma	CRPC	21,7	WNR	ADT, Chemotherapy
P165_2				38,2		
P167_1	56	Adenocarcinoma	CRPC	593,2	WNR	ADT, Abiraterone
P167_2				327,5		Enzalutamide

P167_3				NA		
P167_4				7570,0		Chemotherapy
P167_5				7605,2		
P169_1	58	Adenocarcinoma	CRPC	595,8	WNR	ADT, Chemotherapy, Abiraterone
P169_2				615,2		Enzalutamide
P169_3				960,4		Chemotherapy
P169_4				1244,4		
P170_1	63	Adenocarcinoma	CRPC	16,0	NA	ADT, Chemotherapy
P170_2				3,5	133,0	Abiraterone
P170_3				2,6	14,2	Chemotherapy
P170_4				3,6	13,8	
P178_1	62	Adenocarcinoma	CRPC	209,1	WNR	RTX, ADT
P178_2				196,1		Abiraterone
P178_3				92,2		
P179_1	64	Adenocarcinoma	CRPC	0,4	59,0	ADT
P179_2				NA	NA	
P179_3						RTX, Chemotherapy
P179_4				0,6	218,0	
P181_1	66	Adenocarcinoma	CRPC	111,0	WNR	Abiraterone
P181_2				NA		
P182_1	52	glandular	CRPC	122,9	WNR	ADT, Abiraterone, Chemotherapy, Enzalutamide
P182_2				194,5		
P182_3						

Supplementary Table 2

List of 74 cancer-related genes for which targeted resequencing was performed.

Genes
AKAP9
AKT1
APC
AR
ARID1A
ASXL1
ATM
AXIN1
BAX
BRAF
BRCA1
BRCA2
BRIP1
CDH1
CDKN2A
CDKN2B
CHEK2
CREBBP
CTNNA1
CTNNB1
DAPK3
EGFR
EP300
ERBB2
FBXW7
FGFR3
FOXA1
GATA3
GATA4
HNF1A
HRAS
KDM6A
FOCAD
KLF6
KMT2D
KRAS
MAP2K4
MDM2
MED12
MLH1
KMT2A

KMT2C
MLLT3
MSH2
MSH6
MUTYH
MYH9
KAT6B
NF1
NOTCH1
NRAS
PALB2
PDE4D
PIK3CA
PIK3R1
PMS1
PMS2
PTCH1
PTEN
RAD51C
RB1
RUNX1
SMAD4
SMO
SPOP
STK11
TMEM135
TP53
TRRAP
UBR5
VHL
ZBTB7A
TMPRSS2
ERG
